# Lactylation prognostic signature identifies DHCR7 as a modulator of chemoresistance and immunotherapy efficacy in bladder cancer

**DOI:** 10.3389/fimmu.2025.1585727

**Published:** 2025-07-15

**Authors:** Yuanqiao Zhao, Zhuo Xing, Yongqi Zhao, Haozhe Xu, Ruilin Liu, Tiejun Yang, Yinhuai Wang, Xuan Zhu

**Affiliations:** ^1^ Department of Urology, The Second Xiangya Hospital, Central South University, Changsha, Hunan, China; ^2^ Department of Urology, The Affiliated Cancer Hospital of Zhengzhou University and Henan Cancer Hospital, Zhengzhou, China

**Keywords:** bladder cancer, lactylation, chemotherapy, Dhcr7, immunotherapy, prognostic signature, tumor immune microenvironment

## Abstract

**Background:**

Bladder cancer (BLCA), the 10th most common cancer worldwide, presents a worsening prognosis as the disease progresses. Reliable tools for predicting BLCA prognosis and treatment efficacy remain urgently needed.

**Methods:**

Expression profiles of lactylation related genes were analyzed utilizing the Cancer Genome Atlas (TCGA) database and BLCA data from the GSE13507 dataset. Two distinct clusters were identified through unsupervised clustering analysis. Lactylation associated gene signatures were established and subsequently validated using training cohort and different validation cohorts. Immune cell infiltration patterns and drug response profiles were systematically evaluated. Parallel analyses of lactylation related genes were conducted at the single-cell resolution. A series of *in vivo* and *in vitro* experiments were subsequently performed to validate the findings.

**Results:**

We examined the mRNA expression profiles of 22 lactylation related genes in BLCA tissues. Through comprehensive analysis, we identified two distinct lactylation clusters that exhibited significantly different clinical outcomes and tumor immune microenvironment characteristics. Building upon these findings, we subsequently stratified patients into two molecular subtypes according to the lactylation clusters and established a robust genetic signature for predicting survival outcomes in BLCA patients. The lactylation risk score showed a strong connection with survival outcomes and correlated with the tumor microenvironment (TME) immunosignature and predicted immunotherapy efficacy. DHCR7 emerged as a pivotal prognostic gene from the nine gene model, prompting subsequent focused analyses. Single-cell analysis confirmed that DHCR7 reached peak expression in tumor epithelial cells, whereas TCGA data and single-cell data demonstrated strong associations between DHCR7 and diverse immune-cell populations. For the first time, we identified that knockdown of DHCR7 enhances the efficacy of both cisplatin chemotherapy and immunotherapy, highlighting DHCR7 as a key player in cisplatin resistance and its influence on immunotherapy effectiveness in BLCA. These findings offer valuable insights into potential combined therapeutic strategies.

**Conclusions:**

We developed a robust lactylation risk prediction model for accurately forecasting BLCA prognosis and identified DHCR7 as a pivotal biomarker involved in cisplatin resistance and influencing immunotherapy efficacy in BLCA.

## Introduction

Bladder cancer is the 10th most common malignancy globally and the second most prevalent urological tumor (1, 2). At diagnosis, 70–75% of patients present with non-muscle invasive bladder cancer (NMIBC), 20–25% with muscle-invasive bladder cancer (MIBC), and 5% with metastatic disease (3). NMIBC management typically involves endoscopic resection and risk based intravesical adjuvant therapy. In contrast, MIBC treatment involves more aggressive approaches, including surgery, radiation, and chemotherapy. Advanced BLCA treatment relies on systemic therapy (3). Despite multiple treatment options and incremental advances in BLCA management, many patients face tumor recurrence even after standardized therapy. Limited sensitivity to current treatments contributes to persistently high overall mortality (4–9). Therefore, identifying novel prognostic biomarkers and predictive tools, along with understanding factors that influence treatment efficacy, particularly for cisplatin-based chemotherapy and immunotherapy, is crucial for advancing personalized and precise treatment strategies for BLCA.

Enhanced glycolysis, one of the most critical metabolic changes during hypoxia, leads to increased lactate production in cancer and participates in various cellular processes (10). Lactate, an abundant tumor metabolite, arises from the Warburg effect within the tumor microenvironment (11). Lactylation involves the covalent attachment of a lactate molecule to a protein through a chemical reaction between lactate and a lysine residue on the protein (12). Lactylation modifications play a crucial role in various biological activities, such as tumorigenesis (13, 14), tumor progression (15), macrophage polarization (16), and drug resistance (17, 18). At present, the relationship between lactylation and BLCA progression, prognosis, immunotherapy, tumor immune microenvironment, and drug resistance remains unclear. Additionally, predictive models for evaluating the prognostic significance of lactylation related genes in BLCA are still lacking. Consequently, exploring the pathological processes, potential biological functions, and effective predictive models of lactylation could provide new strategies for the diagnosis and treatment of BLCA.

In this study, we utilized bulk RNA transcriptome and single-cell RNA sequencing data, integrating various algorithms such as Consensus Clustering, immune infiltration analysis, enrichment analysis, and predictive modeling of lactylation related genes. The stability and reliability of the prognostic model were validated in external cohorts to comprehensively examine the expression patterns of lactylation related genes in BLCA. Among the modeled genes, DHCR7 was identified as significantly overexpressed in BLCA, strongly associated with prognosis, and implicated in regulating the tumor immune microenvironment. Subsequently, a series of *in vivo* and *in vitro* experiments revealed that DHCR7 knockdown enhances the efficacy of both cisplatin chemotherapy and immunotherapy.

## Materials and methods

### Bulk RNA-seq data acquisition and preprocessing

We retrieved BLCA transcriptomic data and clinical profiles from The Cancer Genome Atlas database (https://www.cancer.gov/ccg/research/genome-sequencing/tcga), our analysis included 412 tumor samples and 19 normal samples. For the IMvigor210 dataset, RNA-seq and clinical information were obtained using the R package IMvigor210CoreBiologies (19). In addition, RNA-seq data and relevant clinical details from BLCA cohorts (GSE13507, GSE19423, GSE32894, GSE48075, GSE48276) were obtained from the Gene Expression Omnibus (GEO) database. GSE13507 contains microarray data of 165 primary BLCA samples, 23 recurrent NMIBC tissues, 58 adjacent tissues and 9 normal bladder samples (20). GSE19423 included microarray gene expression analysis of 48 patients with primary pT1 BLCA who received BCG immunotherapy (21). GSE32894 contains the gene expression profile data of 308 cases of urothelial carcinoma (22). GSE48075 included 142 cases of primary BLCA, among which 73 cases were MIBC (23). GSE48276 contains the gene expression profile analysis of 116 cases of urothelial carcinoma (24). Six cisplatin-related datasets (GSE165767, GSE235066, GSE15372, GSE77515, GSE33482, GSE45553) were obtained from the GEO database. GSE165767 presents the gene expression difference map of BLCA cell line T24 with or without cisplatin treatment (25). The GSE235066 dataset collected transcriptome sequencing data of RT112 and 5637 BLCA cell lines treated with or without cisplatin (26). GSE15372, GSE33482 and GSE45553 contain microarrays of expression of normal and cisplatin-resistant ovarian cancer cell lines (27, 28). The transcriptome sequencing data of breast cancer cells treated with cisplatin were obtained in the GSE77515 dataset (29). In the GEO dataset, genes were matched to probes based on platform annotations. For genes corresponding to multiple probes, the maximum expression value was used. In these high throughput experiments, we used R software for corresponding processing, using the Combat function from the sva R package to remove batch effects (30). The lactylation associated genes were identified in previous studies (31). The above datasets were used for the construction and validation of the prediction model and for the analysis of cisplatin sensitivities related genes.

### Single-cell RNA sequencing analysis

Three single-cell sequencing datasets (GSE135337, GSE130001, GSE129845) were obtained from the GEO database, comprising 9 tumor samples and 4 normal tissue samples. Standardized single-cell RNA sequencing (scRNA-seq) data from these BLCA patients were analyzed using the R package Seurat (32). The following cells were excluded: 1) mitochondrial gene expression exceeding 10%; 2) fewer than 200 feature genes; and 3) more than 4000 feature genes. To eliminate batch effects, data integration was performed using the Harmony R package (33). Five algorithms—AUCell, UCell, singscore, ssgsea, and AddModuleScore were applied to single-cell data for enrichment score. CellCall (34), a toolkit that utilizes KEGG pathway-based ligand-receptor-transcription factor (L-R-TF) axis datasets, was employed to infer intercellular communication networks and internal regulatory signals by integrating intra and intercellular signals. Using CellCall R package, we further elucidated specific pathways between DHCR7 high and low expression group cells.

### Consensus clustering and differential gene expression

Unsupervised cluster analysis was conducted using the R package ConsensusClusterPlus to identify unique patterns of genes associated with lactylation. Expression profiling data of lactylation related genes were used to classify patients for further analysis. To ensure classification reliability, 100 replications were performed.

### Model development

To construct a model based on lactylation related genes, we conducted a series of analyses, including cluster differential analysis, univariate Cox regression, and least absolute shrinkage and selection operator (LASSO) regression with ten-fold cross-validation using the glmnet R package (35). For LASSO regression, we selected lambda.min to prevent overfitting. A final set of nine genes (DHCR7, P4HB, CD109, FADS1, HOXC8, CLDN5, TMC7, KRT4, ADIRF) were identified to construct a prognostic formula termed ‘Riskcore’ Risk $score=\;\sum_{i=1}^{n}Coe_{i}\cdot Exp_{\left\{i\right\}}$ , where $Coe_{i}$ represents the coefficients of the genes and $Exp_{\left\{i\right\}}$ represents the relative expression of genes in the cohort.

### Kaplan-Meier survival analysis and tumor mutational burden

Kaplan-Meier (K-M) analysis was conducted to compare survival between high and low groups. The predictive accuracy of Riskscore for 1-, 3-, and 5-year survival was evaluated using ROC curves generated by the timeROC R package (36). We also investigated the expression levels of immune checkpoint genes and tumor mutational burden (TMB) to evaluate their potential as predictive markers for immunotherapy response. BLCA mutation data was retrieved from the TCGA database, and tumor mutational burden was calculated using the maftools R package (37).

### Functional enrichment analysis

Functional enrichment analyses were conducted using the R package clusterProfiler (38), focusing on Gene Ontology (GO) and Kyoto Encyclopedia of Genes and Genomes (KEGG) pathways. Genomic Variation Analysis (GSVA) was conducted using the GSVA R package to compare pathway activation between different groups (39). Gene set enrichment analysis (GSEA) was performed using the R software package. To obtain a comprehensive overview of the proteins translated by each group of differential genes, we utilized the Proteomaps database (40).

### Analysis of tumor microenvironment cell infiltration

The relative abundance of various cells in the tumor was determined using the CIBERSORT and ssGSEA (Single Sample Gene Set Enrichment Analysis) methods, implemented through the CIBERSORT and GSVA R packages, respectively. Using the ESTIMATE tool (41), this study analyzed BLCA gene expression data to estimate stromal content, tumor purity, and immune cell infiltration in cancerous tissues, predicting immune scores, stromal scores, and tumor purity in BLCA.

### Prediction of drug sensitivity

Drug sensitivity analysis was performed using data from the Genomics of Drug Sensitivity in Cancer 2 (GDSC2) database (https://www.cancerrxgene.org/). The relationship between DHCR7 expression and drug sensitivity was analyzed using the oncoPredict R package (42).

### Cell culture

The T24(#SC0113), J82(#SC0116), and MB49(#SC0512) BLCA cell lines were procured from Yuchi Biology (Shanghai, China). They were cultured in DMEM or RPMI-1640 media (Gibco, USA) at 37°C in 5% CO2, respectively. These media contained 10% fetal bovine serum (AC03L055, Shanghai Lifei Lab Biotech, China) and 1% penicillin-streptomycin. All cells were subjected to STR authentication. Additionally, Mycoplasma contamination was checked regularly using a Mycoplasma detection kit (Biotool, Houston, TX).

### Antibodies and reagents

β-actin (#20536-1-AP, Proteintech, 1:6000 dilution), cleaved caspase-3(#19677-1-AP, Proteintech,1:1000 dilution), DHCR7(#ab103296, Abcam, 1:1000 dilution). The following chemicals and reagents were used: water for injection (WFI) for Cell. Culturesodium (ThermoFisher, A1287301) lactate (L-lactate, #867-56-1, MedChemExpress), Oxamate (#565-73-1, MedChemExpress) and Cisplatin (#S1166, Selleck).

### Plasmids and transfection

The short hairpin RNAs (shRNAs) of DHCR7 was obtained from GeneCopoeia (Guangzhou, China). The sequences as followed: human: 5′-GATCCCCTGACTTCTGCCATAAGTTCTCGAGAACTTATGGCAGAAGTCAGGGTTTTTG -3′; mouse: 5′-GATCCCACAGATTTCTGCCAGGTTACTCGAGTAACCTGGCAGAAATCTGTGGTTTTTG -3′ For gene knockdown experiments, cells were cultured in plates or dishes to undergo starvation treatment with serum-free Opti-MEM medium (Gibco, USA) for 12 hours. Then transfected with 2 μg/ml of indicator vector, after 72 hours, cells are collected for further experiments. All transfections were performed using Lipofectamine 2000 (Invitrogen, America) according to the manufacturer’s instructions. After puromycin selection, we obtained cells stably transfected with the indicator plasmids.

### Cell proliferation assay

The cells were seeded in 96-well plates; approximately 10^4^ cells were seeded per well. After culturing for 24 h at 37°C in 5% CO2, the cells were divided into several groups with different treatments. Each group had at least 3 repetitions. Ten microliters of CCK-8 reagent (#C0037, Beyotime, China) were added to each well and incubated for 1 h under the above conditions. The absorbance at 450 nm was measured by a microplate reader. The CCK-8 assay was applied to measure the half maximal inhibitory concentration (IC50) of cisplatin after treatment with a serial dose of cisplatin for 24 h in T24 and J82 cells.

### Apoptosis assay

Caspase-3 activity and Annexin V-FITC/PI assays were used to assess cell apoptosis. The caspase-3 activity assay was performed using the Caspase-3 Assay Kit (ab39401, Abcam) according to the manufacturer’s protocol. For the Annexin V-FITC/PI assay, cells were stained with Annexin V-FITC and PI using the Annexin V-FITC Apoptosis Detection Kit (A21102, Vazyme), following the manufacturer’s instructions. After staining, cells were incubated at room temperature for 15 minutes and analyzed using a flow cytometer. Data analysis was performed with FlowJo software.

### Western blot

Cells were lysed with RIPA buffer containing protease and phosphatase inhibitors (#P0013, Beyotime, China). Protein concentration was determined using a Micro BCA Protein Assay Kit (Sigma-Aldrich). Equal amounts of protein were resolved via sodium dodecyl sulfate-polyacrylamide gel electrophoresis (SDS-PAGE) and transferred onto polyvinylidene fluoride (PVDF) membranes with 0.45 μm pores (Millipore, Bedford, MA, USA). The membranes were blocked with 5% skimmed milk and incubated with primary antibodies overnight at 4°C. The next day, membranes were incubated with secondary antibodies and visualized using enhanced chemiluminescence (ECL) reagent (Sigma-Aldrich).

### RT-qPCR

Total RNA (1 μg) was extracted using Trizol reagent (#AG21102, Accurate Biotechnology, Hunan, China) according to the manufacturer’s instructions. RNA concentration and quality were assessed using a NanoDrop 2000 spectrophotometer (Thermo Fisher Scientific). cDNA was then synthesized using a gDNA-free reagents kit (#AG11728, Accurate Biotechnology, Hunan, China). Reverse transcription quantitative PCR (RT-qPCR) was performed using SYBR Premix ExTaq (Cat. No. AG11701, Accurate Biotechnology, Hunan, China). The primer sequences are provided in **Supplementary Table 1**. GAPDH was used as the internal control for sample normalization. The results are presented as expression levels relative to the control group, which was set to 1.

### Mice study

All animal procedures were approved by the Ethics Committee of the Second Xiangya Hospital, Central South University (Approval No. 20241117). Six-week-old C57BL/6 mice were purchased from Shulaibao Biotechnology (Wuhan, China). MB49 cells (1×10^7^in 100 µl 1×PBS) infected with shControl or shDhcr7 lentivirus were injected s.c. into the right flank of mice. After the xenografts reached a size of approximately 50 mm^3^, mice carrying similar types of tumors were randomized into different groups and treated with anti-PD-1(BioXcell, Clone RMP1-14)/IgG (BioXcell, Clone 2A3) (200 μg, i.p., given at days 0, 3, 6). Mouse was euthanized when it meets the end-point standard required by ethics committee. And the tumor was collected for immune-fluorescence.

### Statistical analysis

All data analyses in the bioinformatics section were conducted using R software (version 4.3.2).

Comparisons between two independent groups were conducted using the two-tailed Wilcoxon test for the raw letter portion unless otherwise specified. All basic experimental data were analyzed using GraphPad Prism V.10, and differences between groups were analyzed using Student’s two-tailed t-test. Spearman correlation analysis was used to evaluate the relationships between variables. Survival differences were assessed using K-M survival curves with log-rank tests. For clarity in presentation, p-values > 0.05 were labeled as ‘ns’, p-values < 0.05 as ‘*’, p-values < 0.01 as ‘**’, and p-values < 0.001 as ‘‘***”. The experimental data were collected from three independent experiments and expressed as mean ± SD. P values below 0.05 indicate statistical significance.

## Results

### Identification of lactylation clusters and differential gene expression in BLCA

**Figure 1:**
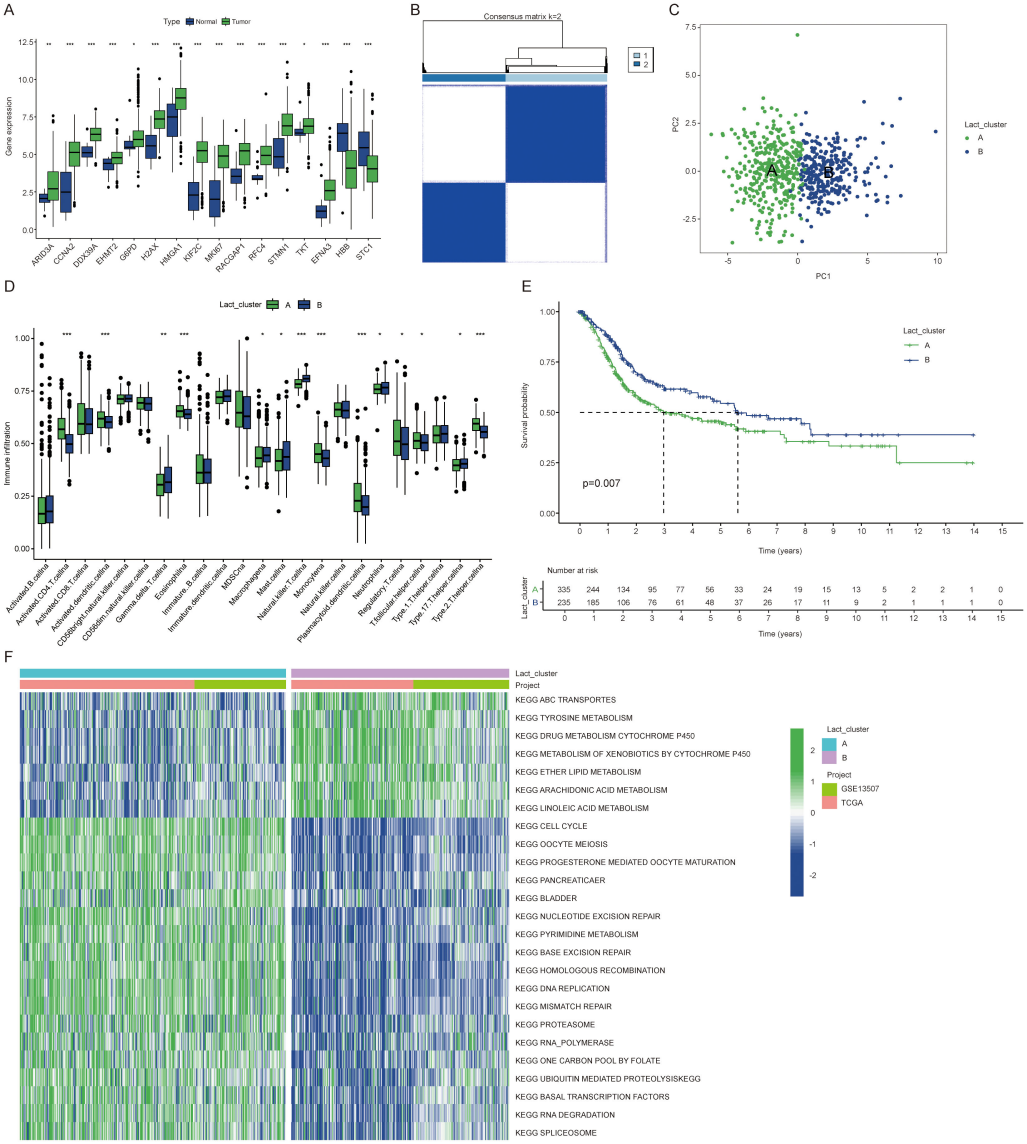
Identification of lactylation clusters and differential gene expression in BLCA. **(A)** Expression differences of lactylation related genes between cancer and normal tissues. **(B)** Consensus matrix showing clustering results with the number of clusters (k) set to 2. **(C)** PCA plot illustrating the two clusters. **(D)** Immune cell expression differences between the clusters. **(E)** K-M curves showing overall survival differences between the two clusters. **(F)** Heatmap showing KEGG enrichment analysis differences between clusters for 25 pathways. The symbols *, **, and *** represent P < 0.05, P < 0.01, and P < 0.001, respectively.

In this study, we examined the expression profiles of lactylation related genes in both normal and tumor samples. Our analysis revealed that genes such as ARID3A, CCNA2, and DDX39A were consistently and significantly overexpressed in cancer tissues (**Figure 1A**). High expression of most lactylation related genes correlates with poorer prognosis (**Supplementary Figures S1A–O**). Using the ConsensusClusterPlus R package, we performed unsupervised clustering of BLCA patients based on lactylation related genes, resulting in the identification of two distinct patient clusters (**Figures 1B, C**). We observed a significant difference in immune cell infiltration between the two patient clusters. Notably, Cluster B exhibited higher levels of infiltrating immune cells, including natural killer T cells (**Figure 1D**). Patients in Cluster B also demonstrated a more favorable prognosis (**Figure 1E**), suggesting that enhanced immune cell presence within the tumor microenvironment may contribute to improved clinical outcomes. In light of the survival differences observed between the two clusters, we conducted a pathway enrichment analysis using the GSVA algorithm. This analysis revealed distinct pathway profiles for each cluster. Specifically, Cluster A exhibited higher enrichment scores for cell cycle, ubiquitin-regulated protein degradation, and RNA degradation pathways. The significant enrichment of these pathways may explain the poorer prognosis associated with Cluster A (**Figure 1F**).

### Identification of gene subtypes influenced by BLCA lactylation clusters

**Figure 2:**
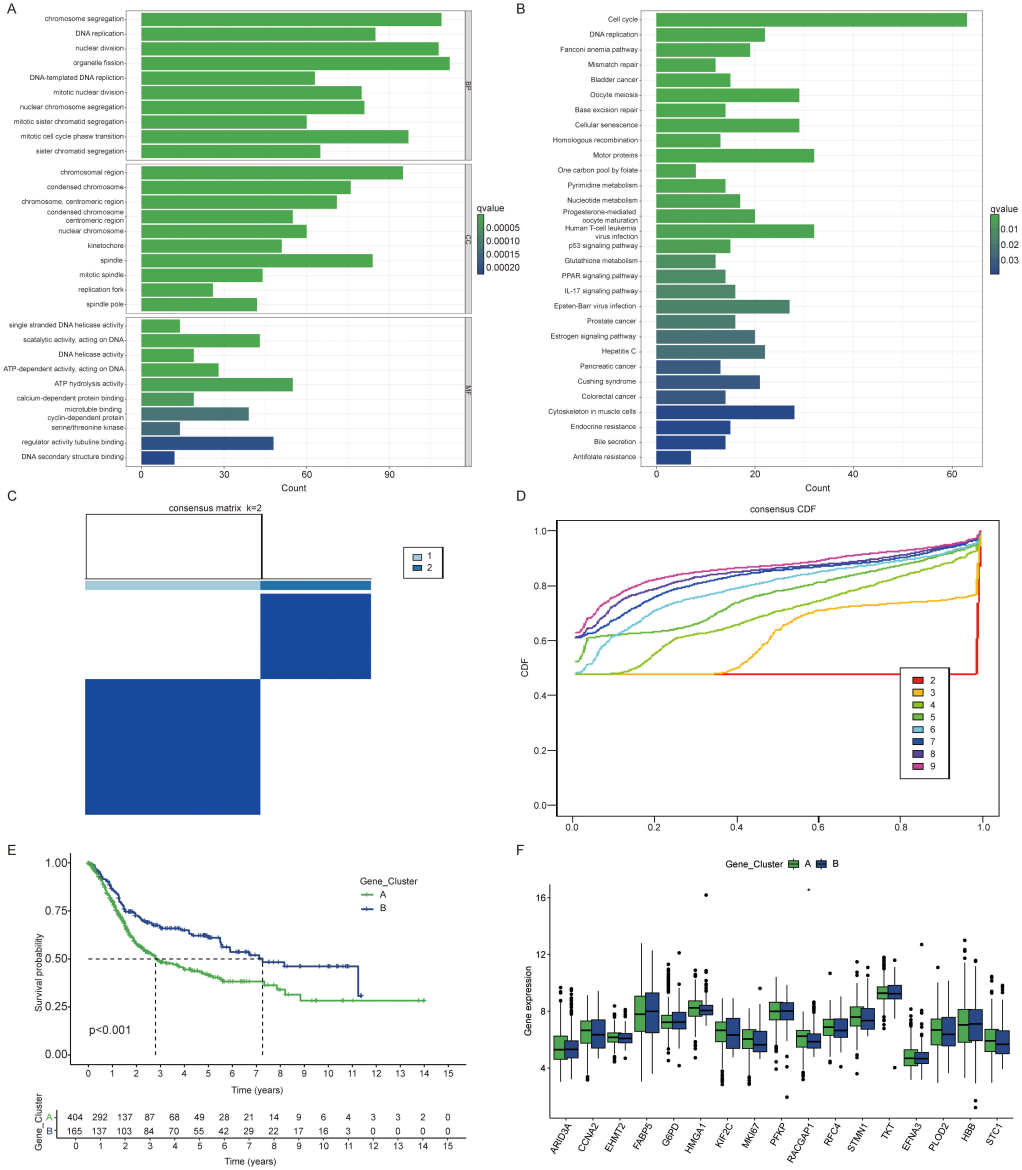
Identification of gene subtypes influenced by BLCA lactylation clusters. **(A)** Gene Ontology (GO) analysis highlighting enriched biological processes (BP), cellular components (CC), and molecular functions (MF) between clusters. **(B)** KEGG enrichment analysis of differentially expressed genes between clusters. **(C, D)** Consensus matrix illustrating clustering results with the number of clusters (k) set to 2. **(E)** Kaplan Meier curves of overall survival differences between two clusters. **(F)** Expression differences of lactylation related genes among gene clusters. The symbols *represent P < 0.05.

Subsequently, we employed the “limma” package to identify 1,250 differentially expressed genes (DEGs) associated with the lactylation clusters. This enabled us to further explore the distinct biological behaviors exhibited by each cluster. We performed functional enrichment analyses, including GO and KEGG pathway evaluations, on the DEGs associated with the lactylation clusters (**Figures 2A, B**). From these differentially expressed genes, we identified 692 genes with significant prognostic associations (p < 0.05) through one-way Cox regression analysis. Patients were grouped into two genetic subtypes based on these 692 prognoses associated genes (**Figures 2C, D**). We observed a significant difference in prognosis between the two gene clusters (**Figure 2E**). Interestingly, RACGAP1 was the only gene that showed a significant difference between the two gene clusters (**Figure 2F**).

### Development and validation of a lactylation related gene signature

**Figure 3:**
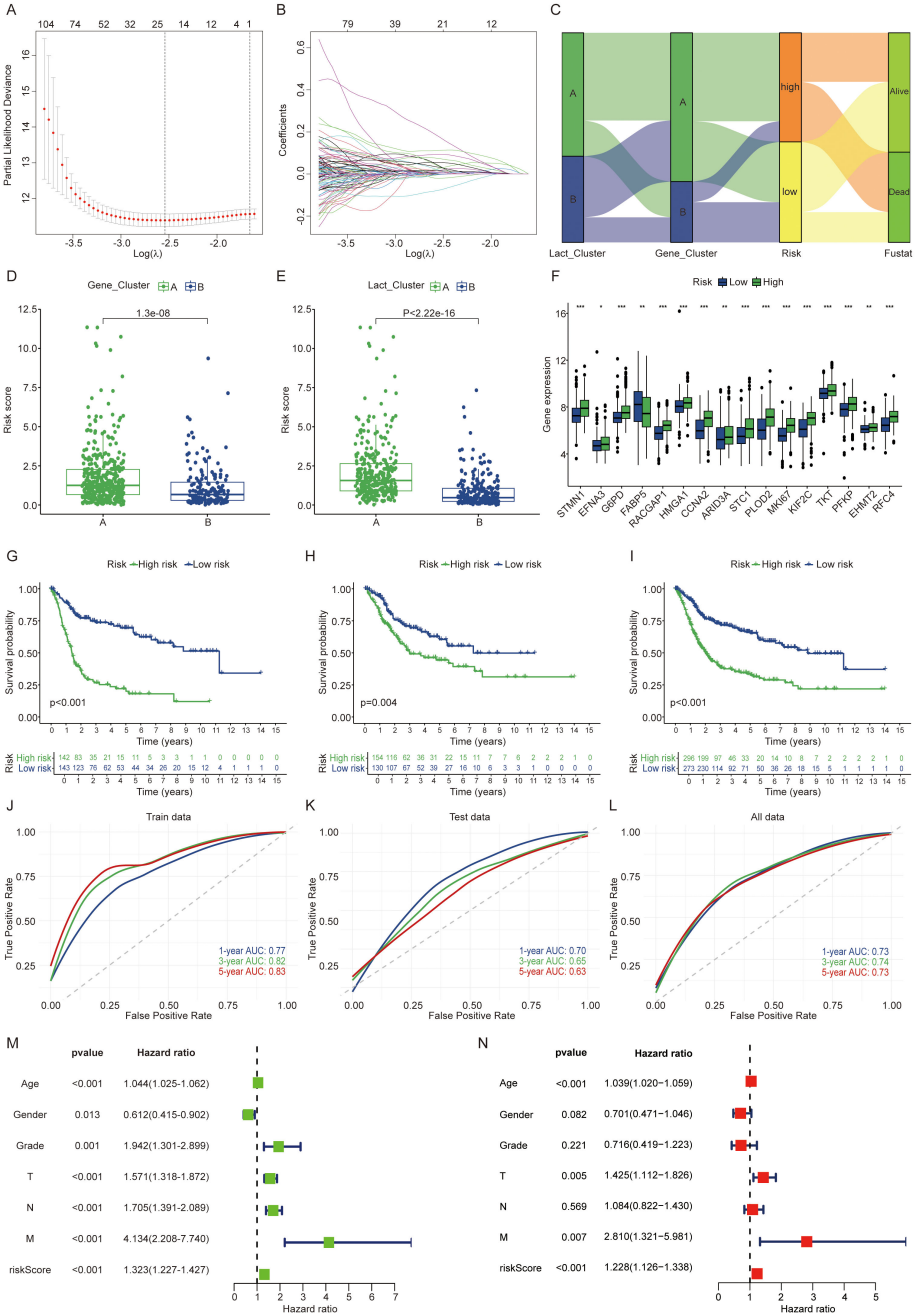
Development and validation of a lactylation-related gene signature. **(A, B)** LASSO regression analysis using the minimal lambda value. **(C)** Sankey diagram linking clusters, risk score groups, and BLCA survival status. **(D, E)** Risk score differences between clusters. **(F)** Lactylation related genes expression differences between risk groups. **(G-I)** Patients with high lactylation risk scores showed worse prognosis in the train, validation and overall groups. **(J-L)** The ROC curves indicate higher model effectiveness. **(M, N)** Univariate and multivariate Cox regression analyses of risk scores and clinical features in the integrated cohort. The symbols *, **, and *** represent P < 0.05, P < 0.01, and P < 0.001, respectively.

All patients were randomly divided into training and validation sets at a 1:1 ratio. From the initial 1,250 differentially expressed genes, 692 were identified as preliminary prognostic genes through one way cox regression analysis. Using the LASSO algorithm, this set was further narrowed down to a final selection of nine genes (**Figures 3A, B**). The final prognostic risk score was derived from nine gene signatures associated with patient prognosis. Prognostic scores were calculated from the expression levels of these genes using the following formula: Riskscore=DHCR7*0.240+P4HB*0.604+CD109*0.128+FADS1*0.216+HOXC8*0.210+CLDN5*0.296+TMC7*(-0.250) +KRT4*0.103+ADIRF*(-0.041). Patients were classified into high or low score groups based on the median Risk score value. To illustrate the relationships among clustering, Risk score subgroups, and survival status in BLCA, we utilized Sankey diagrams (**Figure 3C**). Cluster A exhibited a consistently poorer prognosis and a higher likelihood of falling into the high-risk group, indicating that patients in cluster A are more prone to adverse outcomes. Patients belonging to the lactylation or gene cluster A exhibited higher risk scores, which aligns with the patterns observed in the Sankey diagram (**Figures 3D, E**). Additionally, we observed that high risk patients exhibited higher expression levels of lactylation related genes (**Figure 3F**). The KM survival curve clearly shows that patients in the high score group have a significantly worse prognosis (P < 0.05) (**Figures 3G–I**). Risk scores demonstrated a worsening trend across clinicopathologic subgroups, with higher risk score groups correlating with poorer prognosis (**Supplementary Figures S3A–N**). High risk patients exhibit an increased risk of death (**Supplementary Figures S4A–C**). In the train patient cohort, we assessed the predictive performance of our prognostic model by generating receiver operating characteristic (ROC) curves for 1-, 3-, and 5-year overall survival (OS) (**Figure 3J**). The area under the curve (AUC) values were 0.77, 0.82, and 0.83, respectively, highlighting the model’s strong predictive accuracy, particularly for long-term survival. Furthermore, the model demonstrated high predictive performance in both the validation and overall cohorts (**Figures 3K, L**). Additionally, we validated the accuracy of the model in three independent external validation datasets (GSE13507, GSE32894, GSE48075) achieving highly satisfactory results with 5-year AUC values of 0.71, 0.89, and 0.69, respectively. In the immunotherapy cohort, we observed that high-risk patients had a worse prognosis following immunotherapy. Similar trends were noted within both the responding and non-responding subgroups (**Supplementary Figures S2H–J**). Besides, elevated expression levels of PDL1, PD1, and CTLA4 were observed in the high risk group (**Supplementary Figures S3O–Q**). Univariate and multivariate Cox regression analyses, incorporating other clinicopathologic factors, demonstrated that the Risk core is an independent risk factor (**Figures 3M, N**). To investigate the biological basis of the prognostic differences between high and low risk groups, we conducted further analyses. GSEA enrichment analysis of differences between high and low risk groups revealed the top five pathways, with significant activation of the cell cycle pathway in the high-risk group (**Supplementary Figure S3L**). The lactylation risk score correlated with tumor microenvironment cells, with a high score indicating increased M2 macrophage infiltration and reduced CD8+ T cells (**Supplementary Figures S4D–I**). This indicates that the high risk group likely contributes to tumor progression by activating cell cycle pathways and inhibiting the tumor immune microenvironment.

### DHCR7 as an important prognostic gene

**Figure 4:**
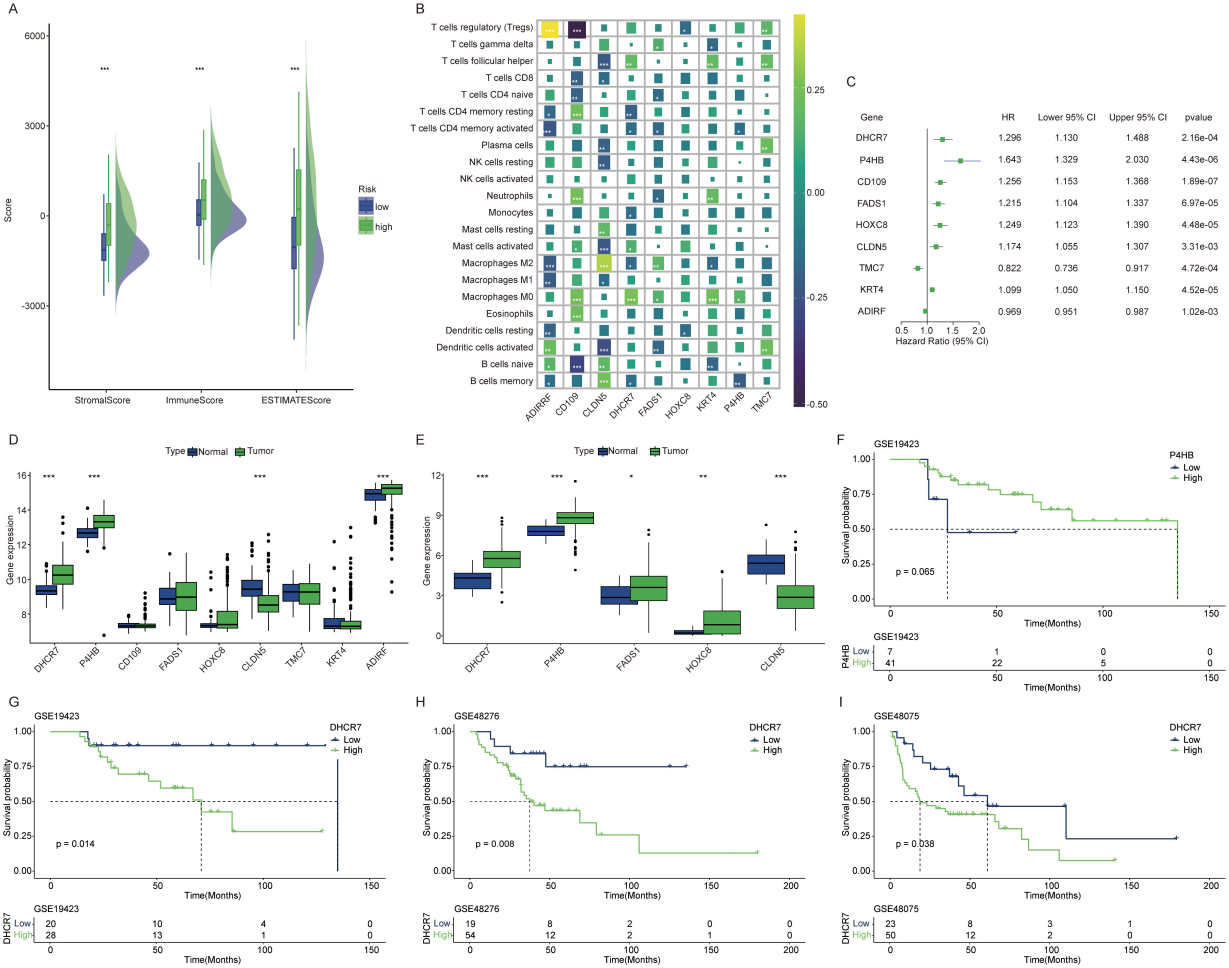
DHCR7 as an important prognostic gene. **(A)** Tumor microenvironment score differences between high and low risk lactylation groups. Green for high risk group, blue for low risk group. **(B)** Correlation analysis between model genes and various immune cells. Different colors represent different correlation coefficients. **(C)** Univariate Cox regression analysis of genes in the model. **(D, E)** Expression differences of model genes between normal and tumor tissues [**(D)** GSE13507 cohort, **(E)** TCGA cohort]. **(F, G)** In the GSE19423 cohort, only DHCR7 showed a significant association with prognosis. **(H, I)** In the GSE48276 and GSE48075 cohort, K-M survival curves comparing DHCR7 High and Low expression groups. The symbols *, **, and *** represent P < 0.05, P < 0.01, and P < 0.001, respectively.

High risk patients exhibit higher stromal scores and immune scores (**Figure 4A**). Given that the high-score group showed increased immune cell infiltration, we next analyzed the correlation between the genes in the constructed model and various immune cell types (**Figure 4B**). Each gene in the model demonstrates a strong correlation with various immune cell types. Recognizing the poorer prognosis of high-risk patients, we further analyzed the relationship between each gene in the model and patient outcomes. A univariate Cox regression forest plot illustrates the prognostic relevance of all genes in the model (**Figure 4C**). For instance, DHCR7, P4HB, CD109, and FADS1 are identified as risk factor genes, whereas TMC7 and ADIRF serve as protective genes. All these genes exhibit p-values below 0.05. In the TCGA cohort and GSE13507 cohort, DHCR7 and P4HB consistently exhibited high expression in tumor tissues (**Figures 4D, E**). In the GSE19423 cohort, only high DHCR7 expression was linked to poorer prognosis (**Figures 4F, G**). In the GSE48276 and GSE48075 cohorts, patients with high DHCR7 expression exhibited poorer prognoses (**Figures 4H, I**). Building on these findings, the analysis will now focus on DHCR7.

### Multi dimensional analysis of DHCR7 in BLCA

**Figure 5:**
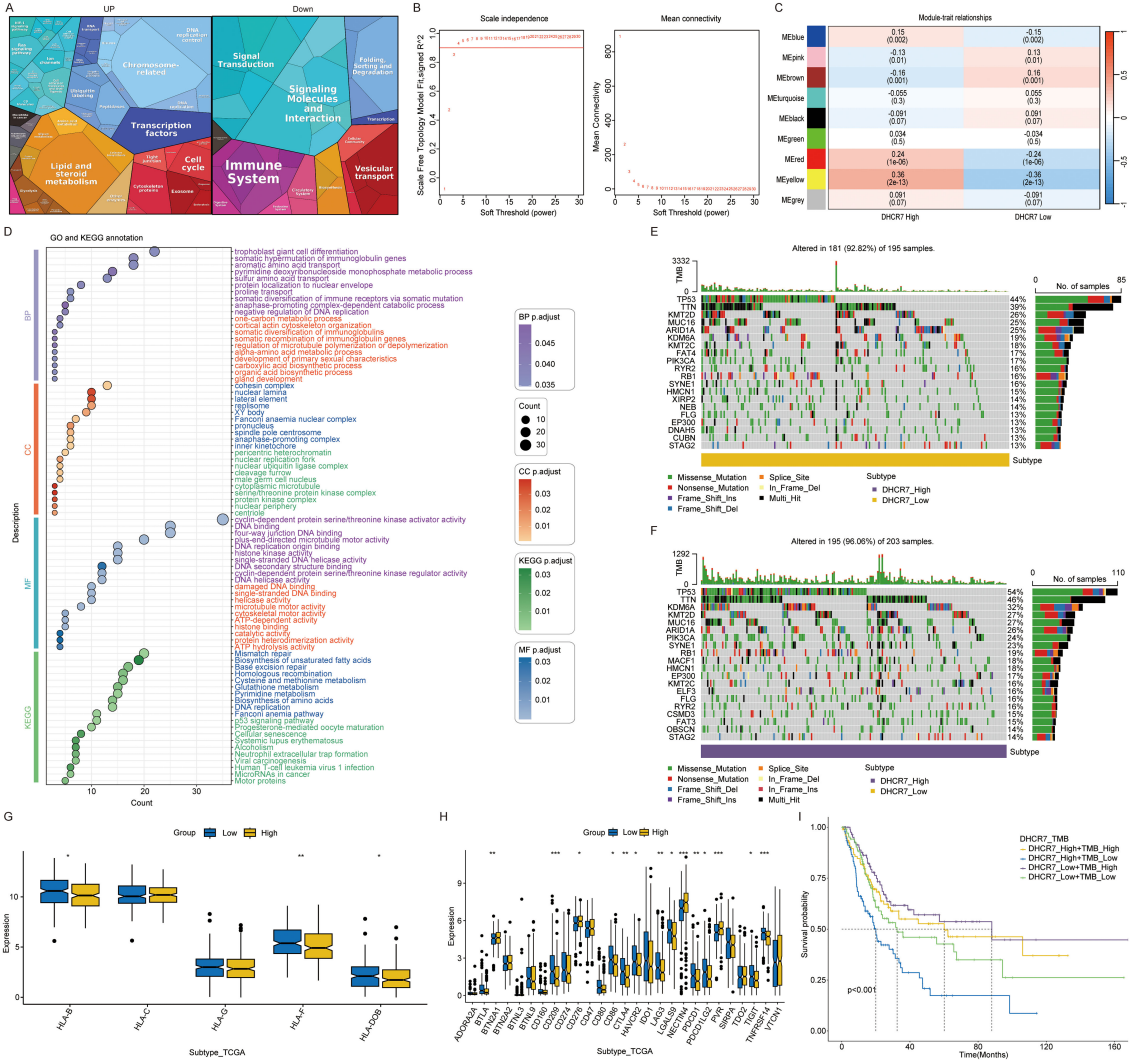
Multi dimensional analysis of DHCR7 in cancer. **(A)** Proteomaps enrichment of DHCR7 high and low expression gene group. **(B)** Selection of soft-thresholding power in WGCNA. **(C)** Module-trait relationships for DHCR7 expression groups. **(D)** GO and KEGG enrichment of yellow module genes. **(E, F)** Mutation landscape in DHCR7 high and low expression groups. **(G)** Differential expression of HLA-related genes between DHCR7 high- and low-expression groups. **(H)** Immune checkpoints and ADC targets show differential expression between DHCR7 expression groups. **(I)** Combined survival analysis of DHCR7 expression and tumor mutation burden (TMB). The symbols, *, **, and *** represent P < 0.05, P < 0.01, and P < 0.001, respectively.

Samples categorized into high and low expression groups based on the median DHCR7 value, with absolute logFC > 0.5, were analyzed for enrichment using Proteomaps. Up-regulated genes show significant enrichment in lipid metabolism, transcription factors, cell cycle, ubiquitination, and chromosome-associated pathways. In contrast, down-regulated genes exhibit significant enrichment in the immune system, signaling molecules, and signal transduction pathways (**Figure 5A**). However, using the WGCNA method, a correlation analysis of the DHCR7 gene was conducted. The yellow gene module was selected for GO and KEGG enrichment analyses, revealing significant associations between DHCR7 and the P53-related pathway, DNA repair, fatty acid synthesis, and glutathione metabolism. These findings suggest that DHCR7 may contribute to tumor progression through these pathways (**Figures 5B–D**). Supplementary figures illustrate the WGCNA analysis process (**Supplementary Figures S5A–C**). We conducted a waterfall plot analysis of gene mutation data. The analysis revealed that TP53 mutations ranked first, with patients exhibiting high DHCR7 expression showing a higher TP53 mutation rate. TTN mutations followed in frequency (**Figures 5E, F**). Boxplot demonstrated significant differences in HLA family gene expression between the DHCR7 high and low expression groups (**Figure 5G**). Specifically, HLA-F and HLA-DOB showed significantly higher expression in the DHCR7 low expression group, indicating potential immune regulatory roles associated with DHCR7 expression levels. BLCA response to immunologic and antibody-drug conjugate (ADC) therapeutic drugs. Next, the correlation between DHCR7 and individual immune checkpoints and ADC target were examined. Boxplot showed that patients with high DHCR7 expression had elevated levels of PVR, NECTIN4, and TDO2, while BTLA and CD209 exhibited reduced expression (**Figure 5H**). TMB plays a pivotal role in tumor biology. Analyzing its relationship with DHCR7, we found that K-M survival analysis stratified patients into four distinct prognostic groups based on combined DHCR7 expression and TMB levels (**Figure 5I**). Patients with high DHCR7 expression and low TMB demonstrated the poorest survival outcomes, while those with low DHCR7 expression and high TMB had the best outcomes, highlighting the prognostic significance of integrating DHCR7 expression and TMB in BLCA. Based on this observation, we hypothesized that DHCR7 could play a role in BLCA immunotherapy.

### Single-cell transcriptomics reveals DHCR7 modulation of tumor microenvironment

**Figure 6:**
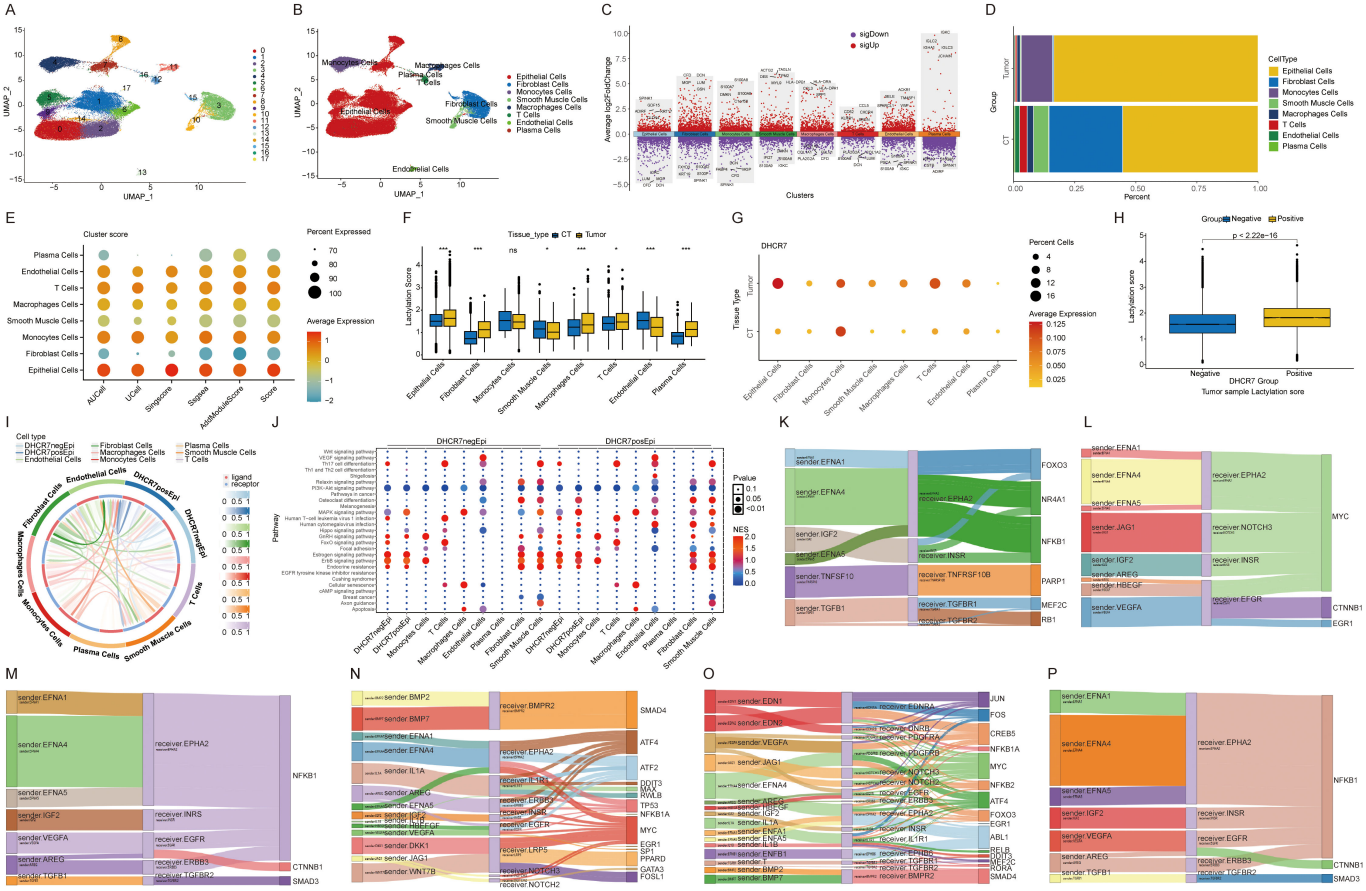
Single-cell transcriptomics reveals DHCR7 modulation of tumor microenvironment. **(A, B)** UMAP plot showing the distribution of 8 main cell types in the integrated dataset. **(C)** Top 5 genes for each cell type. **(D)** Cellular composition ratio chart. **(E, F)** Analysis of lactylation enrichment score differences. **(G)** Dot plot of DHCR7 expression across cells and tissue types. **(H)** Lactylation score differences between DHCR7 groups. **(I, J)** Cellular communication between epithelial cells and other cells in the DHCR7-positive or negative group. **(K-P)** Cellular communication in the DHCR7 positive group: DHCR7-positive epithelial cells on the left, other cells in the center, and transcription factors on the right. [**(K)** Macrophages Cells, **(L)** Monocytes Cells, **(M)** T Cells, **(N)** DHCR7 positive epithelial, **(O)** Smooth Muscle Cells, **(P)** Plasma Cells] The symbols ns, *, and *** represent not significant, P < 0.05, and P < 0.001, respectively.

The advent of single-cell technology has significantly advanced our understanding of the tumor immune microenvironment. After applying quality control and dimensionality reduction, we analyzed 55,951 cells, which were further classified and annotated into eight distinct clusters (**Figures 6A, B**). The supplementary figure presents the manually annotated result map for single-cell analysis (**Supplementary Figure S6A**). Differential gene expression across distinct cell types (**Figure 6C**). Notably, the proportions of these cell clusters differed markedly between normal and tumor tissues, highlighting the heterogeneity of the tumor immune microenvironment (**Figure 6D**). Heatmap showing enrichment analysis results for intercellular Hallmark gene sets in cancer tissues (**Supplementary Figure S6B**). We observed elevated glycolysis levels in epithelial cells and fibroblasts within tumor tissues. We applied five algorithms to analyze the enrichment of lactylation associated genes. Overall, epithelial cells exhibited the highest lactylation levels (**Figure 6E**). Across tissues, most cells in tumor samples showed elevated lactylation levels, with the exception of smooth muscle cells, which displayed lower levels (**Figure 6F**). These findings suggest that lactylation plays a critical role in tumorigenesis and development. At the bulk level, DHCR7 exhibited high expression in tumor tissues. Additionally, we also observed that DHCR7 was significantly overexpressed in tumor epithelial cells (**Figure 6G**). The analysis above highlighted the significant role of DHCR7 in BLCA. To further investigate its interaction with other cells in the tumor microenvironment, tumor epithelial cells were classified into DHCR7 positive and DHCR7 negative groups based on DHCR7 expression. The higher level of lactylation score in the DHCR7 positive expression group also suggests a potential relationship between DHCR7 expression and lactylation (**Figure 6H**). To further explore the relationship between DHCR7 epithelial positive cells and other cell types, we performed cellular communication analysis in tumor samples. The analysis revealed that DHCR7 positive epithelial cells interacted with various other cells through distinct pathways, driven by different transcription factors (**Figures 6I–P**, **Supplementary Figures S6C, D**). A potential relationship between DHCR7 positive epithelial cells and other cells was observed at the single-cell level. To further investigate, K-M survival analyses were performed to assess the combined prognostic impact of DHCR7 expression and various immune cell subpopulations, including CD8+ T cells, NK cells, macrophages, and CD4+ T cells. Among these, The DHCR7 low + T cells CD8+ high group consistently displayed the best prognosis, highlighting a synergistic effect of reduced DHCR7 expression and elevated CD8+ T cell infiltration on survival. Conversely, the DHCR7 high + T cells CD8+ low group showed the worst outcomes, emphasizing the pivotal influence of DHCR7 expression and immune cell composition on patient prognosis. These results highlight the potential interplay between DHCR7 expression and immune cell mediated tumor microenvironment in shaping patient outcomes (**Supplementary Figures S6E–O**).

### DHCR7 inhibition augments response to anti-PD1 therapy

**Figure 7:**
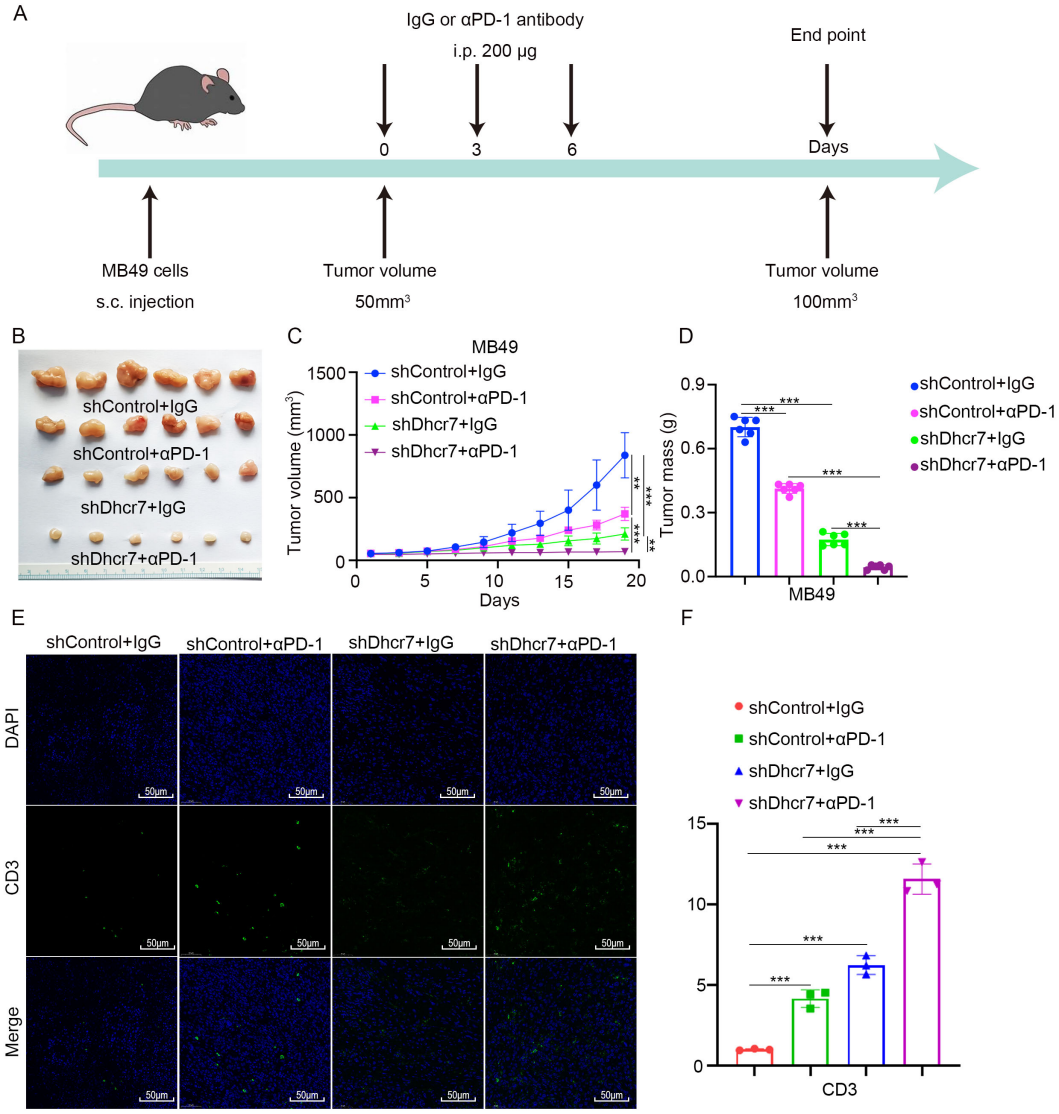
DHCR7 inhibition enhances sensitivity to anti-PD-1 therapy. **(A-D)** MB49 cells were infected with lentivirus vectors expressing control or Dhcr7 shRNAs. These cells were infected with shControl or shDhcr7 and subcutaneously injected into the right dorsal flank of C57BL/6 mice. Mice with subcutaneous MB49 tumors (n = 6/group) were treated with anti‐PD‐1 (200 µg) or nonspecific IgG three times as shown in the schematic diagram **(A)**. The image of the tumor is shown in panel **(B)**. The tumor growth curve was demonstrated in panel **(C)**. Tumor volumes are shown in panel **(D)**. **(E, F)** At the end of treatment, the tumors excised from the mice were dissociated and tumor cells were harvested for immunofluorescence staining. The symbols **, and *** represent P < 0.01, and P < 0.001, respectively.

Immune checkpoint inhibitors have extended survival in advanced BLCA patients, yet their efficacy remains constrained, with many patients developing resistance to immunotherapy (43). This underscores the urgent need to identify the underlying causes of immunotherapy resistance. Based on the following findings: Patients with high lactylation risk scores exhibited poorer responses to immunotherapy. In TCGA data, genes in the low DHCR7 expression group showed activation of the immune system in Proteomaps. Patients with low DHCR7 expression and high TMB demonstrated better prognoses. At the single-cell level, interactions between DHCR7-positive epithelial cells and T cells were observed. Additionally, TCGA data revealed that patients with low DHCR7 expression and high CD8+ T cells infiltration had improved survival outcomes. DHCR7 is not only related to lactoylation, but also participates in cholesterol synthesis. Previous studies have linked elevated cholesterol levels to increased PD-L1 expression, facilitating immune escape (44). Another recent study revealed that DHCR7 significantly influences the tumor microenvironment, with high cholesterol levels contributing to CD8+ T cell exhaustion (45). Based on the above result, we hypothesized that DHCR7 might influence BLCA immunotherapy and that its knockdown could enhance sensitivity to anti-PD-1 treatment. To test this, we conducted *in vivo* experiments and confirmed that Dhcr7 knockdown significantly suppressed tumor growth. When combined with anti-PD-1 treatment, tumor suppression was further enhanced (**Figures 7A–D**). Immunofluorescence analysis showed that Dhcr7 knockdown markedly elevated CD3 expression and the combination with anti-PD-1 treatment amplified this effect (**Figures 7E, F**). These findings suggest that DHCR7 influences tumor immunotherapy sensitivity by modulating the tumor immune microenvironment.

### Role of DHCR7 in cisplatin sensitivity

**Figure 8:**
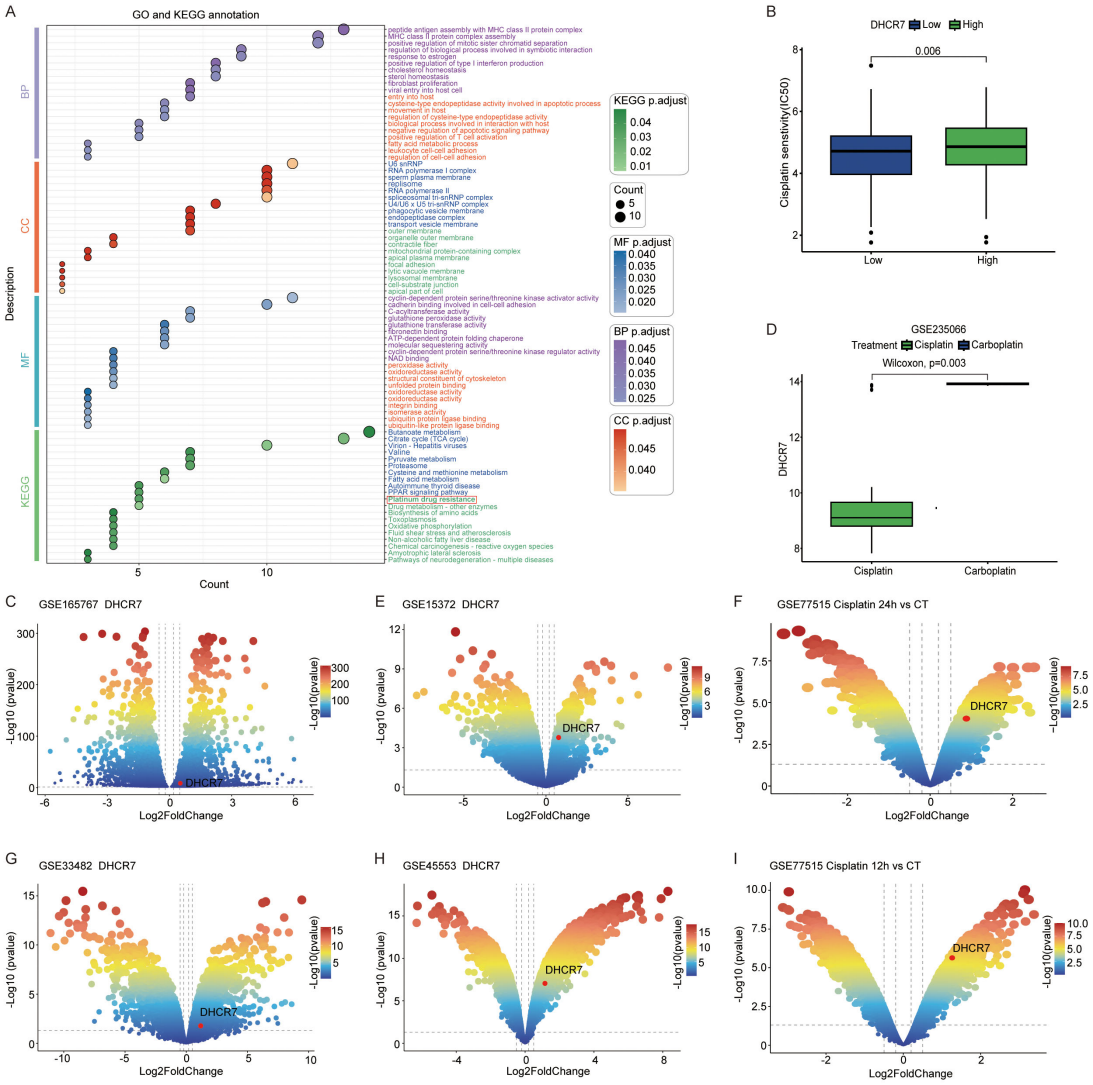
Role of DHCR7 in cisplatin sensitivity. **(A)** GO and KEGG enrichment of differential genes between epithelial DHCR7 groups at the single-cell level in BLCA. **(B)** Cisplatin IC50 values across DHCR7 groups in BLCA. **(C)** Volcano plot of cisplatin resistance in BLCA (GSE165767). **(D)** DHCR7 expression changes across chemotherapeutic agents in BLCA (GSE235066). **(E-I)** DHCR7 involvement in cisplatin-resistant volcano plots of other cancers. [**(E)** Ovarian Cancer, **(F)** Breast Cancer, **(G)** Ovarian Cancer, **(H)** Ovarian Cancer, **(I)** Breast Cancer].

We analyzed the differences between DHCR7 positive and DHCR7 negative epithelial cells in tumor tissues and identified highly expressed genes for GO and KEGG enrichment analyses. Interestingly, the analysis revealed enrichment in pathways related to cisplatin resistance. This finding led us to hypothesize that DHCR7 may contribute to cisplatin resistance in BLCA (**Figure 8A**). Using the oncoPredict R package, we further analyzed DHCR7 and found that patients with high DHCR7 expression had higher IC50 values (**Figure 8B**), indicating reduced sensitivity to cisplatin. In BLCA data (GSE165767), we also observed the involvement of DHCR7 in cisplatin resistance (**Figure 8C**). Additionally, cisplatin showed greater sensitivity compared to carboplatin in one dataset, further complicating the relationship between DHCR7 expression and drug response (**Figure 8D**). To investigate whether this phenomenon extends beyond BLCA, we analyzed other tumors and found that DHCR7 was highly expressed in cisplatin-resistant groups across various cancers (**Figures 8E–I**). These findings strongly suggest that DHCR7 is likely involved in cisplatin resistance in BLCA and potentially in other tumors. Next, we aim to experimentally validate the role of DHCR7 in cisplatin resistance in BLCA and determine whether its involvement in resistance is associated with lactylation.

### Elevated lactylation levels reduce sensitivity to cisplatin therapy in BLCA

**Figure 9:**
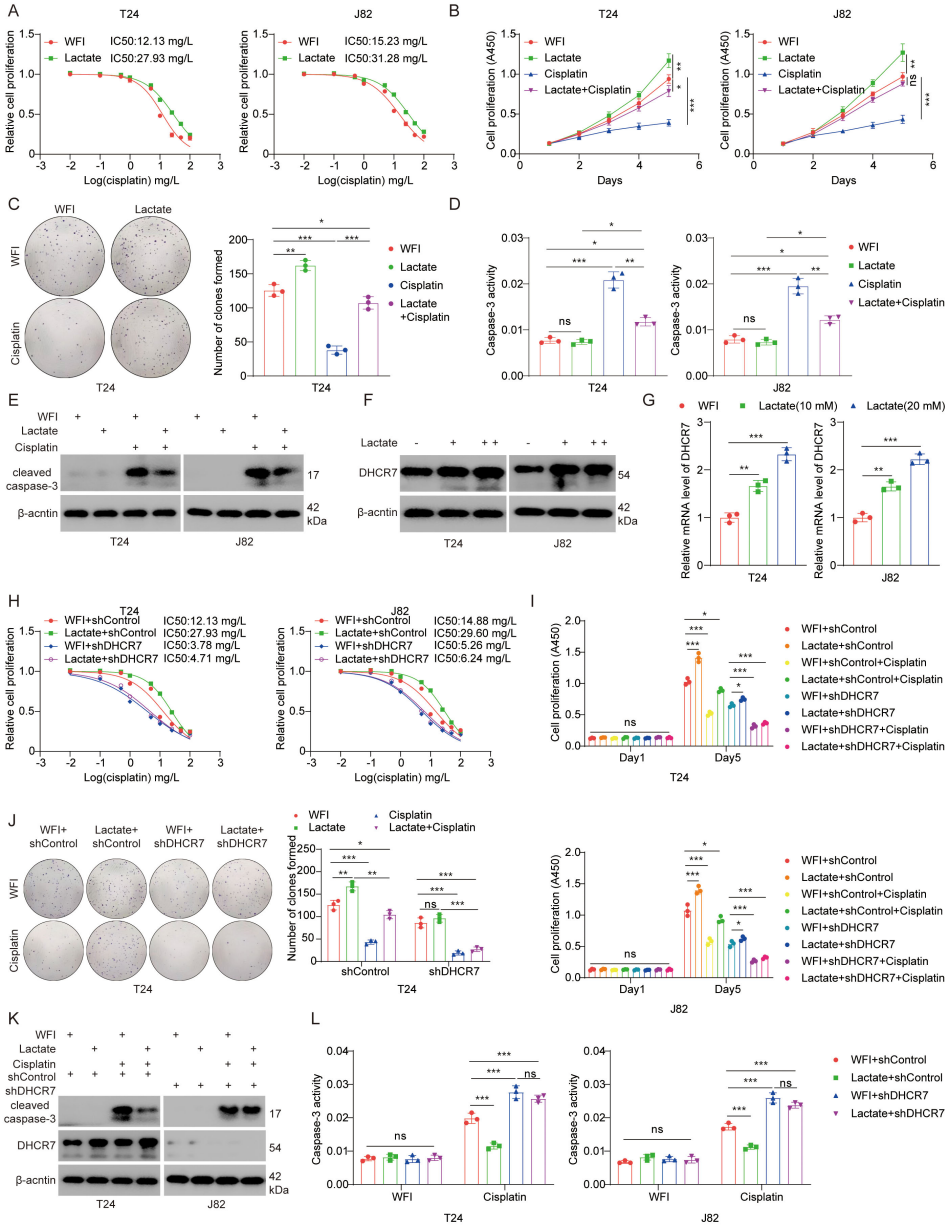
Elevated lactylation levels reduce sensitivity to cisplatin therapy in BLCA. **(A)** T24 and J82 cells were treated with lactate (10 mM) for 24 hours, followed by cisplatin at varying doses for another 24 hours. CCK-8 assays were performed to measure cisplatin IC50 values. **(B)** T24 and J82 cells were treated with lactate (10 mM) for 24 hours, followed by collection for the CCK-8 assay. Data are presented as the mean ± SD from three independent experiments. **(C)** T24 cells were treated with or without lactate (10 mM) for 24 hours, followed by treatment with or without cisplatin (1.0 nM) for an additional 24 hours. The cells were then collected for a colony formation assay. Data are presented as the mean ± SD from three independent experiments. **(D, E)** T24 and J82 cells were treated with lactate for 24 hours, followed by treatment with or without cisplatin for an additional 24 hours. The cells were then collected for caspase-3 activity assay **(D)** and Western blot analysis **(E)**. **(F, G)** T24 and J82 cells were treated with varying doses of lactate for 24 hours. The cells were then collected for Western blot analysis **(F)** and qPCR analysis **(G)** to measure DHCR7 expression levels. **(H)** T24 and J82 cells were transfected with the specified shRNA for 72 hours. After puromycin selection, the cells were treated with varying doses of cisplatin for 24 hours and then subjected to CCK-8 assays to determine cisplatin IC50 values. **(I)** T24 and J82 cells were transfected with the specified shRNA for 72 hours. After puromycin selection, the cells were treated with lactate for an additional 48 hours, followed by treatment with or without cisplatin. The cells were then subjected to CCK-8 assays. **(J)** T24 cells were transfected with the specified shRNA for 72 hours. After puromycin selection, the cells were treated with or without cisplatin and lactate, and then subjected to colony formation or CCK-8 assays. Data are presented as the mean ± SD from three independent experiments. **(K, L)** T24 cells were transfected with the specified shRNA for 72 hours. After puromycin selection, the cells were treated with or without cisplatin and lactate. The cells were then collected for Western blot analysis **(K)** and caspase-3 activity assay **(L)**. Data are presented as the mean ± SD from three independent experiments. The symbols *, **, and *** represent P < 0.05, P < 0.01, and P < 0.001, respectively.

Our results demonstrated that lactate treatment of BLCA cells increased the median inhibitory concentration (IC50) and reduced their sensitivity to cisplatin (**Figure 9A**). Conversely, treatment with Oxamate resulted in a reduction in IC50 (**Supplementary Figure S7A**). Indicating a role for lactate in modulating cisplatin resistance. Colony formation and CCK-8 assays demonstrated that lactate promotes tumor progression and attenuated the sensitivity of BLCA cells to cisplatin (**Figures 9B, C**). Oxamate treatment led to decreased cell proliferation (**Supplementary Figure S7B**). As expected, lactate reduces the pro-apoptotic effect of cisplatin on BLCA cells (**Figures 9D, E**), whereas oxamate enhanced this effect (**Supplementary Figures S7C, D**). Then, how does lactete regulate DHCR7 and affect the sensitivity to cisplatin treatment? Our results showed that lactate upregulated DHCR7 expression at both protein and mRNA levels (**Figures 9F, G**). Conversely, oxamate inhibited the expression of DHCR7 (**Supplementary Figures S7E, F**). The IC50 curve showed that DHCR7 knockdown significantly increased the sensitivity of BLCA cells to cisplatin and reversed the effect of lactate on cisplatin sensitivity (**Figure 9H**). DHCR7 knockdown increased cisplatin sensitivity in cell proliferation (**Figures 9I, J**). Similarly, down-regulation of DHCR7 promotes apoptosis and enhances the sensitivity of BLCA cells to cisplatin (**Figures 9K, L**). In conclusion, lactylation-associated DHCR7 reduces cisplatin sensitivity in patients with BLCA.

## Discussion

Significant progress has been achieved in the diagnosis and treatment of BLCA. However, the survival rate remains unsatisfactory, hindered by challenges such as metastasis, recurrence, and drug resistance. Recent studies have identified lactylation modification as a critical factor in cancer progression, drug-resistant and metastasis (17, 46, 47). Li et al. revealed the association between histone lactylation and cisplatin resistance in BLCA through single-cell transcriptome analysis. Their research showed that cisplatin-resistant BCa cells exhibit intracellular lactate accumulation and increased levels of histone H3K18 lactylation (H3K18la). H3K18la activates the expression of YBX1 and YY1, inducing cisplatin resistance in BCa (48). Similarly, Deng et al. demonstrated that H3K18la regulates PRKN-mediated mitophagy, promotes M2 macrophage polarization, and facilitates immune escape in BCa (49). Xie et al. showed that CircXRN2 inhibits H3K18la-driven tumor progression by activating the Hippo signaling pathway in human BLCA (50). These findings suggest that histone lactylation may play an important role in BLCA progression. Beyond histone lactylation, recent studies have indicated that numerous non-histone proteins can also undergo lactylation modification and play critical roles in BLCA progression. A study by Xing et al. confirmed that increased lactylation levels of YTHDC1 suppress the sensitivity of BLCA to enfortumab vedotin treatment (51). Jin et al. revealed that mannose inhibits PKM2 lactylation, induces pyroptosis in BLCA, and activates anti-tumor immune responses (52). Given the pivotal role of lactylation in BLCA development, this study aims to investigate the biological significance of lactylation-related genes in BLCA, identify potential therapeutic targets, and provide novel insights for BLCA treatment.

Our results showed that the lactylation risk score characterization model performed well in the BLCA cohort and three independent external GEO validation sets and showed similar effectiveness in other tumors (53, 54). Cluster A patients exhibited higher risk scores, poorer prognoses, and distinct differences in the immune microenvironment. The prognostic utility of risk score was further validated in an immunotherapy cohort. Analysis of single-cell data revealed that lactylation related genes play a significant role in the tumor microenvironment. As previously reported, these genes may contribute to tumor progression and immunotherapeutic response by regulating the tumor immune microenvironment (55). Our study revealed that lactylation risk score associated DHCR7 knockdown enhances the response to BLCA immunotherapy, aligning closely with findings from a recent study in Glioblastoma multiforme (45). The primary function of DHCR7 involves regulating genes associated with cholesterol synthesis. At the single-cell level, DHCR7 showed high expression in macrophages, T cells, and endothelial cells. Additionally, DHCR7-positive epithelial cells interacted with various other cells through distinct pathways. Dong et al. found that the elevated cholesterol level of macrophages in glioma can promote the growth of tumor cells and inhibit the anti-tumor effect of CD8+ T cells (45). In our study, prognostic analysis combining DHCR7 with different immune cells revealed that DHCR7 might play a role in modulating the tumor immune microenvironment. Notably, patients with low DHCR7 expression and high CD8-positive T cell infiltration exhibited the best prognosis, while those with low DHCR7 expression and low M2 macrophage infiltration also had favorable outcomes. Another study indicates that DHCR7 contributes to M2 macrophage polarization in hepatocellular carcinoma, promoting tumor growth and metastasis (56). These findings suggest that lactylation associated genes may contribute to tumor progression not only through tumor cells but also by altering the tumor immune microenvironment via interactions with other cells.

We identified DHCR7 as significantly overexpressed in tumors and involved in cisplatin resistance. Zeng et al. indicated that DHCR7 is involved in the progression of BLCA through the cholesterol pathway (57). Kanmalar et al. confirmed that increased cholesterol synthesis plays an important role in the sensitivity of cisplatin treatment for BLCA (58). In addition, sequencing data revealed that DHCR7 knockdown leads to the downregulation of ENO2, a key glycolysis gene (59, 60). Previous studies have shown that histone lactylation promoting YTHDF2 expression (13), and the DHCR7 is regulated by YTHDF2 (57). This suggests that DHCR7 may be indirectly influenced by lactylation through multiple pathways. Elevated DHCR7 may, in turn, upregulate ENO2 and ENO2 contributes to lactate accumulation, forming a vicious cycle that drives BLCA progression. At the single-cell level, DHCR7 positive epithelial cells displayed higher lactylation scores compared to DHCR7 negative cells. *In vitro* experiments further demonstrated that lactate increased DHCR7 expression, supporting our hypothesis. However, a more detailed exploration of the underlying mechanisms requires further investigation in future studies.

Some studies suggest that chemotherapy and immunotherapy yield better outcomes in BLCA (61–63); however, certain patients still experience suboptimal responses. Our study offers new perspectives on BLCA treatment. In patients with markedly elevated DHCR7, DHCR7 inhibitate effectively enhance the efficacy of combined chemotherapy and immunotherapy. Despite the robustness of our predictive model, several limitations should be acknowledged. First, this study primarily relies on retrospective data, necessitating validation through prospective cohort studies. Second, while we demonstrated that DHCR7 mediates cisplatin resistance in BLCA, the precise molecular mechanisms require further investigation. Thirdly, while the tumor immune microenvironment of BLCA remains highly complex, our study has only provided an initial investigation into the immunotherapeutic role of DHCR7. Further research is required to elucidate the detailed mechanisms underlying DHCR7’s involvement in the BLCA immune microenvironment.

## Conclusion

Our study offers valuable insights into the expression patterns and roles of lactylation related genes in BLCA. Lactylation risk score emerges as an effective predictive tool, capable of forecasting prognosis and immunotherapy efficacy, serving as a guide for precision medicine. Additionally, we found that DHCR7 mediates cisplatin resistance in BLCA, and its knockdown significantly enhances immunotherapy efficacy. This study offers a novel therapeutic approach for BLCA patients with limited response to cisplatin-based combination immunotherapy.

## Data Availability

The datasets presented in this study can be found in online repositories. The names of the repository/repositories and accession number(s) can be found in the article/**Supplementary Material**.
