# Supplementary material for: Lactylation prognostic signature identifies DHCR7 as a modulator of chemoresistance and immunotherapy efficacy in bladder cancer
Source: Front Immunol. 2025 Jul 15;16:1585727. doi: 10.3389/fimmu.2025.1585727 (PMC12303948; doi:10.3389/fimmu.2025.1585727)
Supplement: Supplementary file 1 [file DataSheet1.pdf]

Lactylation prognostic signature identifies DHCR7 as a modulator of chemoresistance and immunotherapy efficacy in bladder cancer

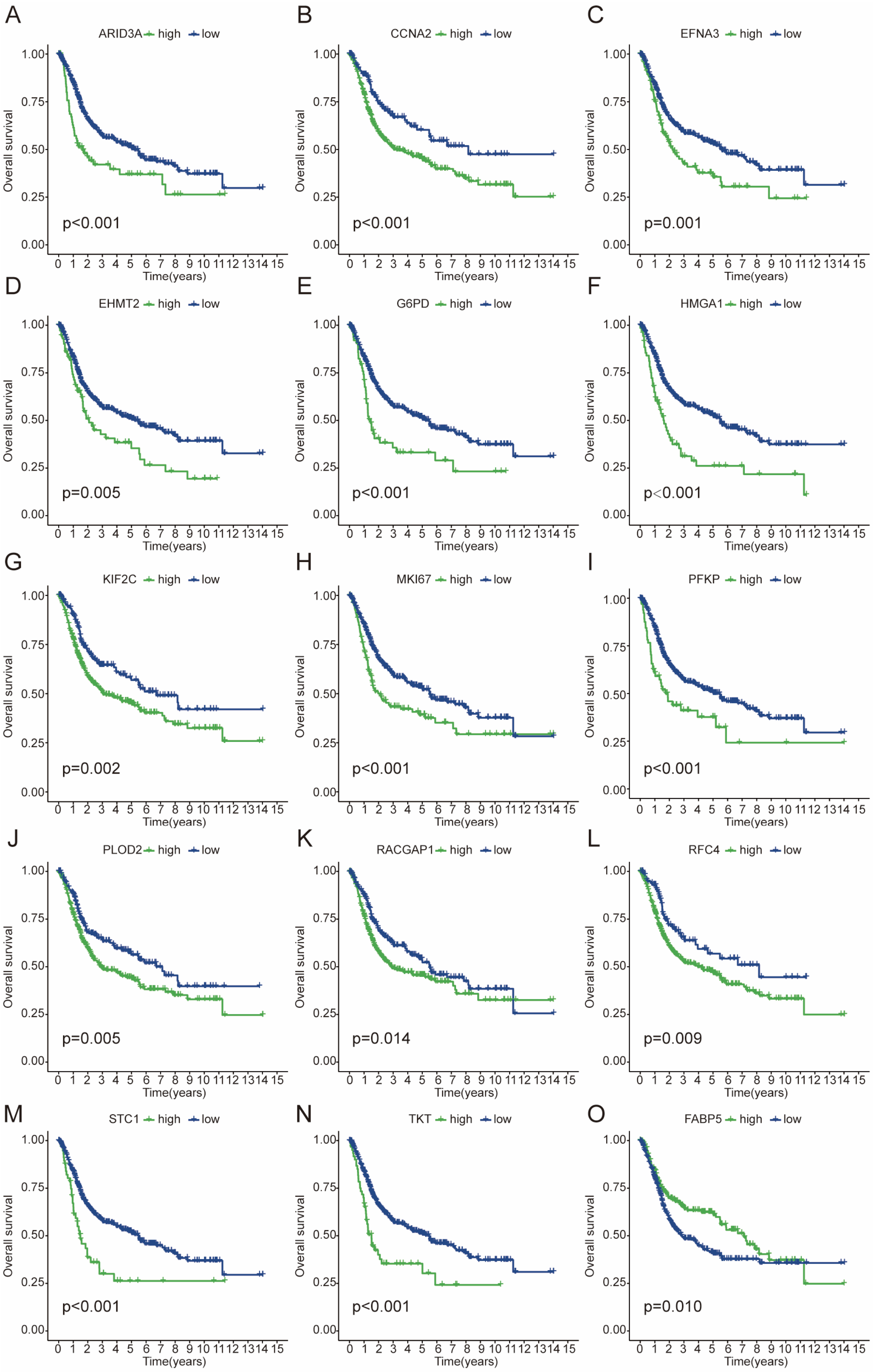

Figure S1. Kaplan-Meier survival curve analysis of various lactylation related genes

(A-N) Higher expression of these lactylation related genes correlates with poorer prognosis. (O) Higher expression of the FABP5 is linked to improved prognosis.

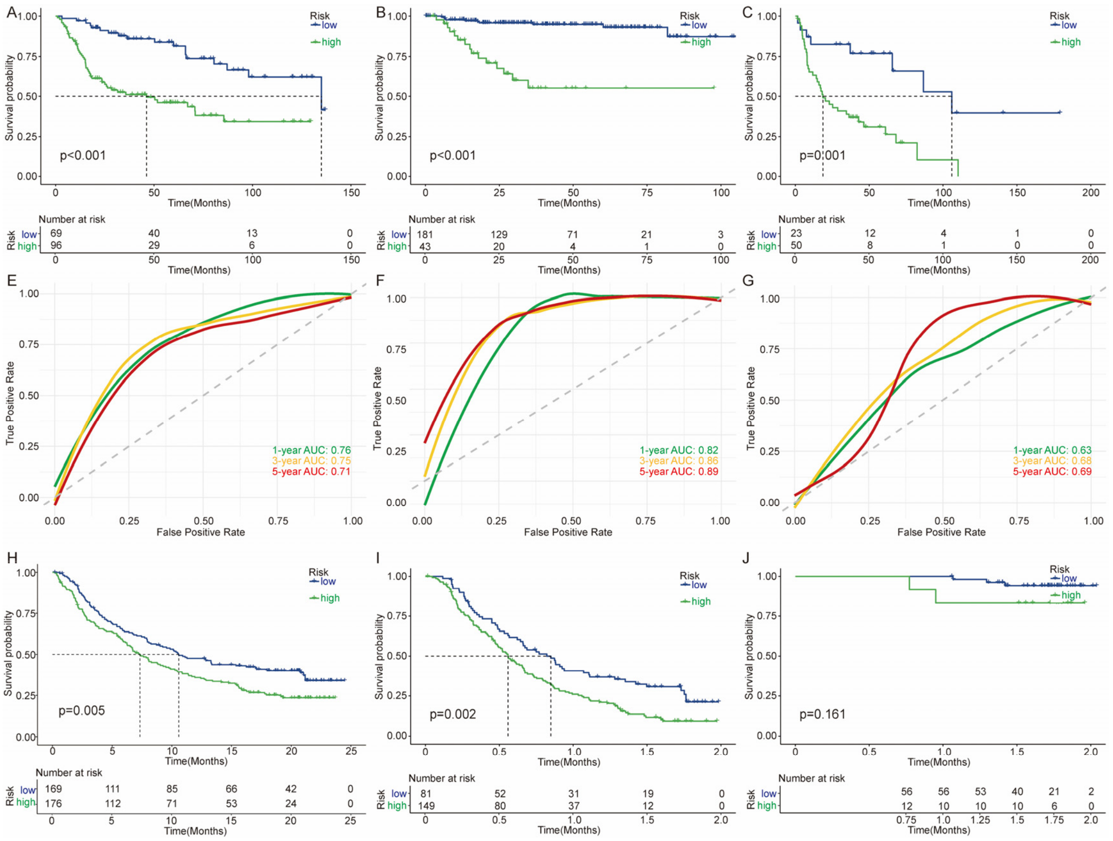

Figure S2. Validation of the lactylation related risk model

(A, E) In the GSE13507 cohort, high risk patients had a worse prognosis, and the model showed superior performance in predicting long term patient outcomes. (B, F) In the GSE32894 cohort, high-risk patients had a worse prognosis, and the model demonstrated improved accuracy in predicting long term outcomes. (C, G) In the GSE48075 cohort, high-risk patients had a worse prognosis, and the model performed well in predicting long term outcomes. (H-J) In the immunotherapy cohort IMvigor210, high-risk patients in the overall population and the SD subgroup exhibited poor prognosis. Although no significant difference was observed in the CR subgroup, a certain trend was evident (SD: 1; CR: J). (SD: Stable Disease, CR: Complete Response)

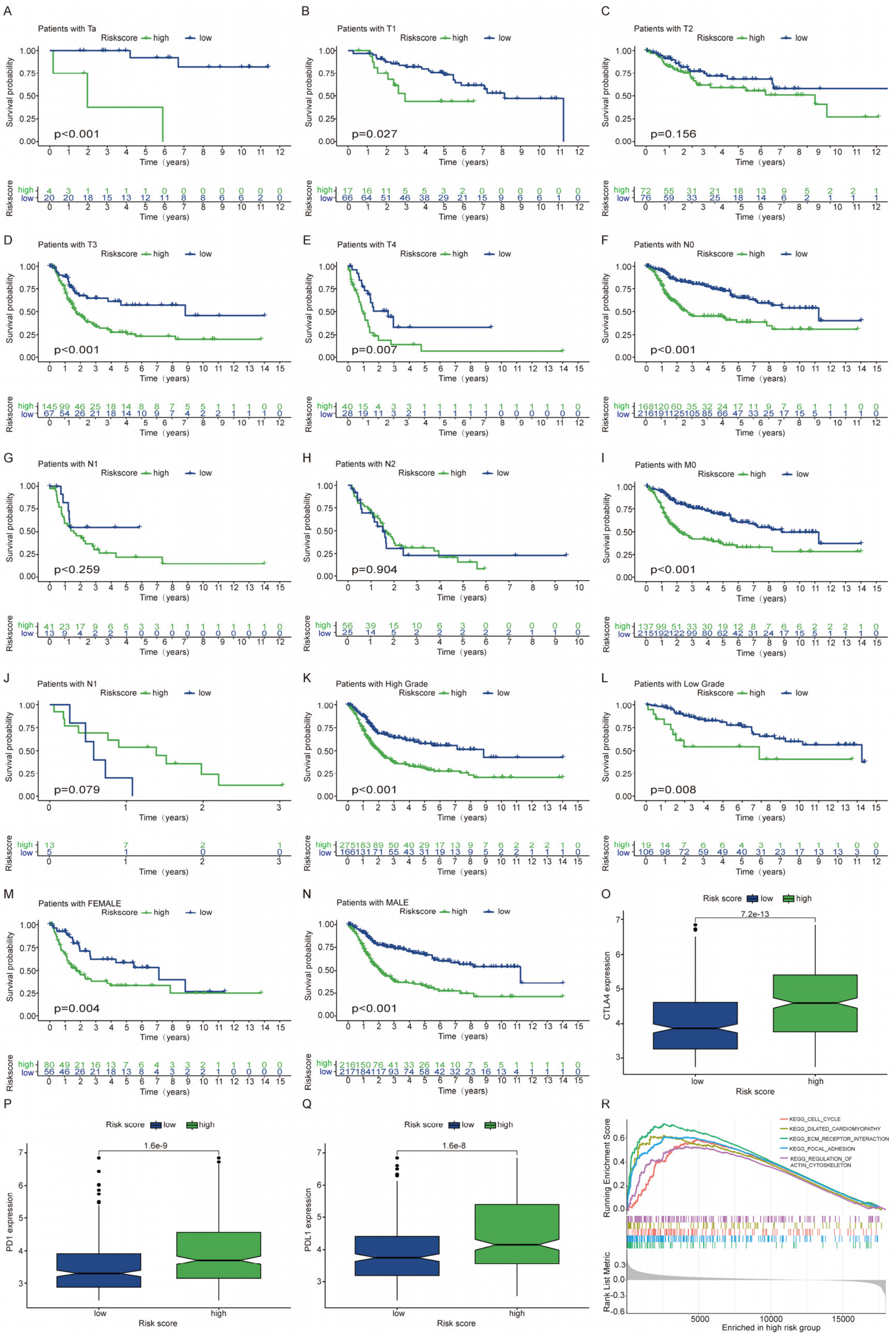

Figure S3. Kaplan Meier survival curves for model scores across various clinical subgroups and GSEA analysis

(A-E) Survival analysis of high and low scores across different T stages. (F-H) Survival analysis of high and low scores across different N-stages. (I-J) Survival analysis of high and low scores across

different M-stages. (K-L) Survival analysis of high and low scores across different Grade. (M-N) Survival analysis of high and low scores across different genders. (O-Q) Immune checkpoint expression in high- and low-risk groups. (R) Top 5 GSEA pathways on high groups.

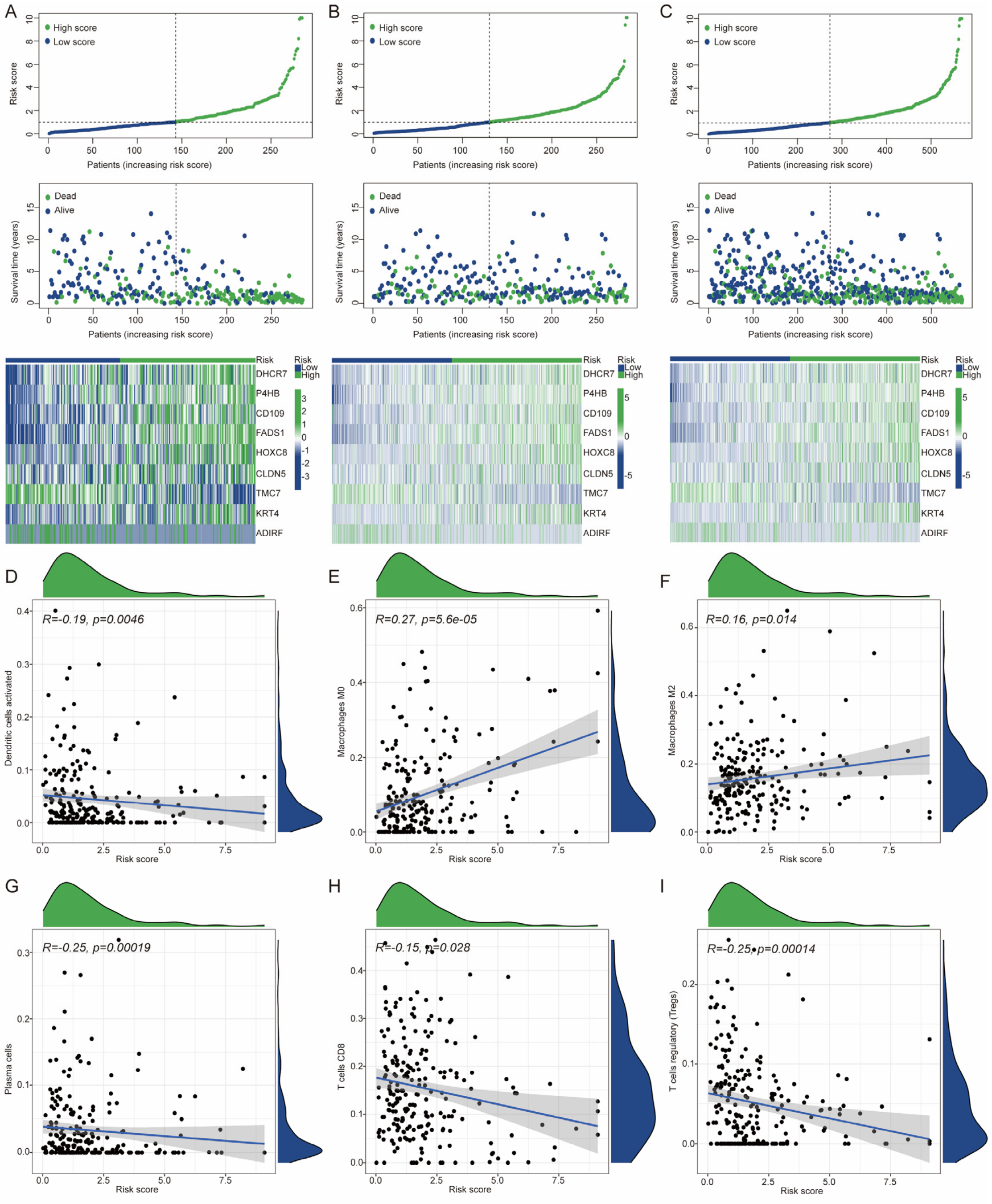

Figure S4. Survival analysis and immune cell correlation

(A-C) Risk score distribution among bladder cancer patients, sorted from lowest to highest. Survival status by risk score for each patient. Heatmap of 9 gene expression levels across risk score groups (sorted top to bottom), train dataset (A), test dataset (B), total dataset(C). (D-I) Correlation analysis of risk scores with each immune cell.

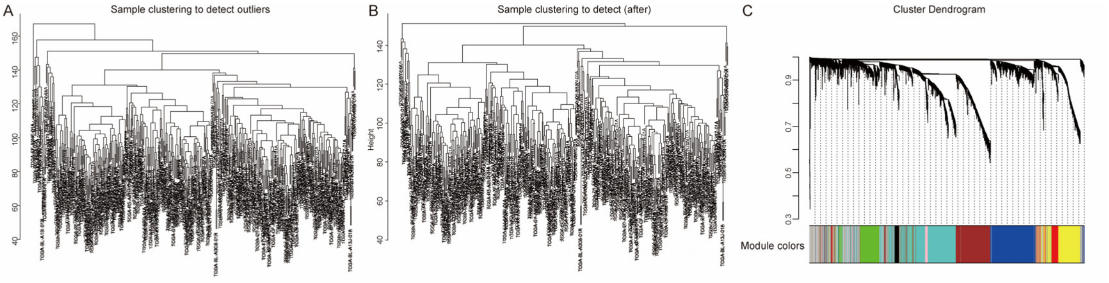

Figure S5. WGCNA analysis in TCGA bladder cancer data

(A-B) Before and after clustering. (C) Hierarchical clustering identified co-expression gene modules, each represented by a unique color. Different colors indicate distinct modules.

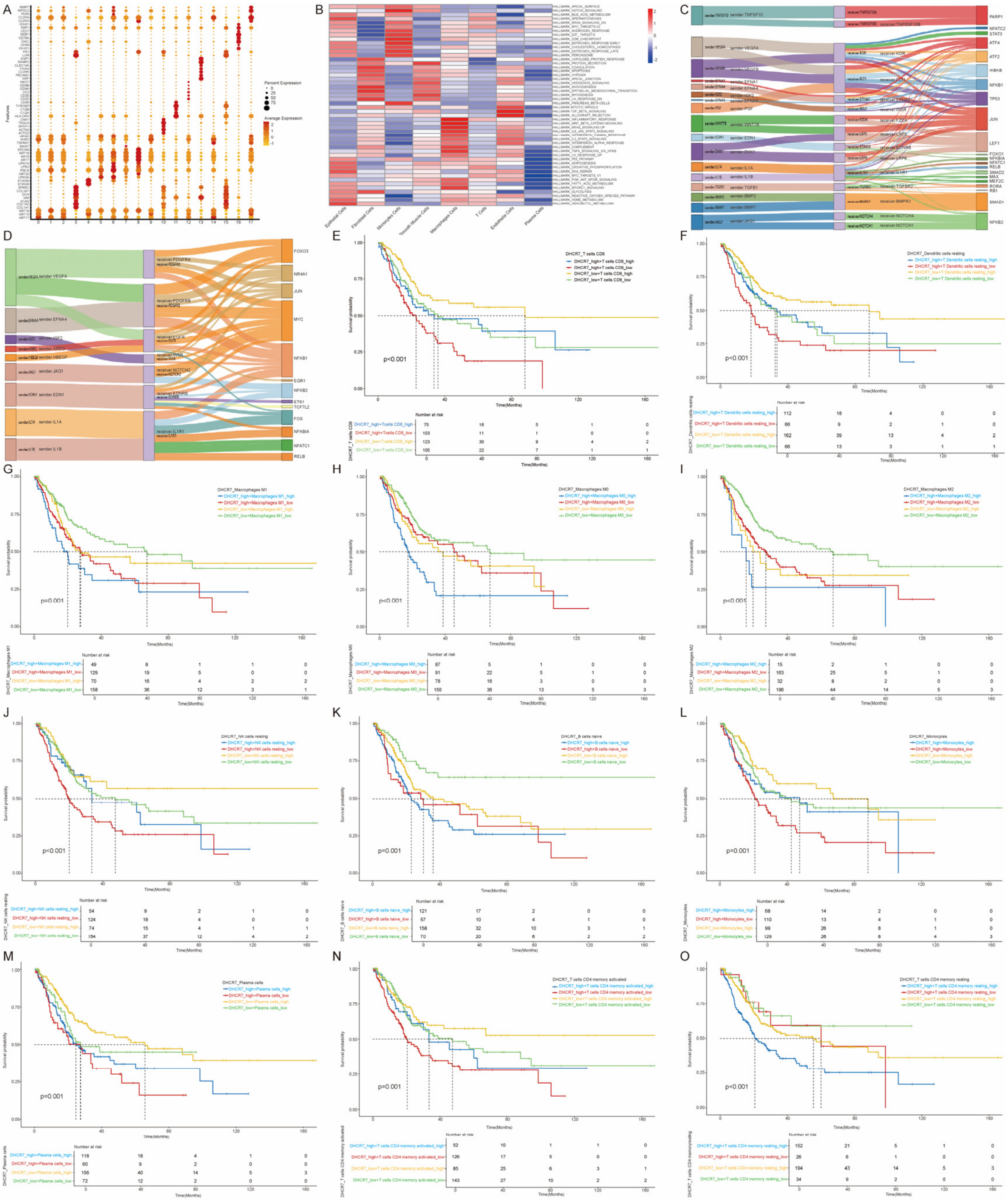

Figure S6. Tumor microenvironment analysis and prognostic evaluation

(A) Single-cell annotation table with marker genes as rows and clusters as columns. (B) Enrichment analysis of 50 pathways in tumor samples using the AddModuleScore function. (C-D) Cellular communication between epithelial cells and other cells in the DHCR7 positive group, with transcription factors displayed on the right (Endothelial Cells: C, Fibroblast Cells: D). (E-O) Combined Kaplan-Meier survival curve of DHCR7 and various immune cells, E:CD8+T cells, F: Dendritic cells resting, G:M1 Macrophage, H:M0 Macrophage, I: M2 Macrophages, J: NK cells resting, K: B cells naïve, L: Monocytes, M: Plasma cells, N: T cells CD4 memory activated, O: T cells CD4 memory resting)

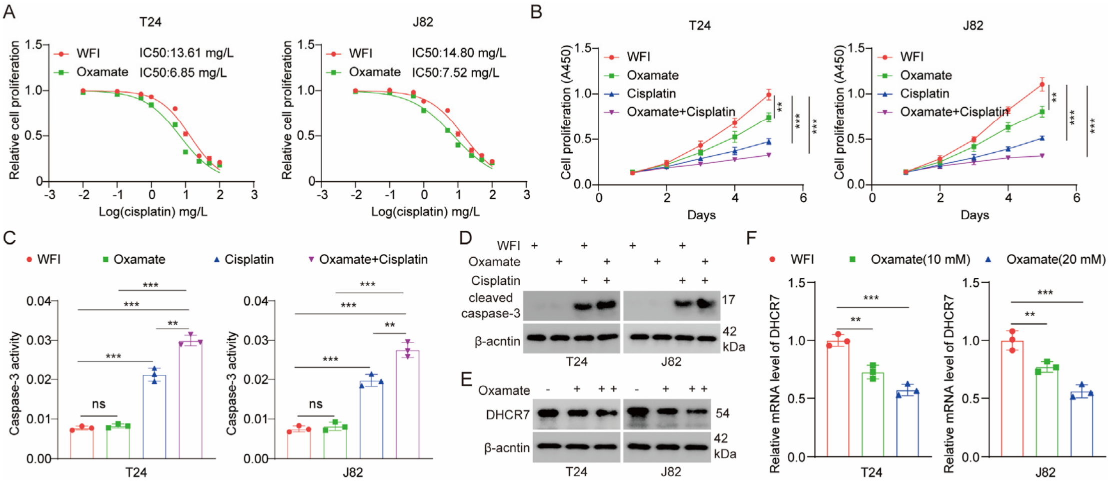

Figure S7. Oxamate mediates cisplatin resistance

(A) T24 and J82 cells were treated with Oxamate for 24 hours, followed by cisplatin at varying doses for another 24 hours. CCK-8 assays were performed to measure cisplatin IC50 values. (B) T24 and J82 cells were treated with Oxamate or cisplatin for 24 hours, followed by collection for the CCK-8 assay. Data are presented as the mean ± SD from three independent experiments. (C-D) T24 and J82 cells were treated with or without cisplatin and Oxamate. The cells were then collected for caspase-3 activity assay (C) and Western blot analysis (D). (E-F) T24 and J82 cells were treated with varying doses of Oxamate for 24 hours. The cells were then collected for Western blot analysis (E) and qPCR analysis (F) to measure DHCR7 expression levels.

Table S1. The primer sequences for RT-qPCR.

Gene （Human）	Forward primer (5′ - 3′)	Reverse primer (5′ - 3′)
GAPDH	GATTCCACCCATGGCAAATTC	CTGGAAGATGGTGATGGGATT
DHCR7	CCCCTGGCTAGAGGGTAGG	TCAACCGGCTAAAGTCCTGC
